# Rosmarinic acid suppresses colonic inflammation in dextran sulphate sodium (DSS)-induced mice via dual inhibition of NF-κB and STAT3 activation

**DOI:** 10.1038/srep46252

**Published:** 2017-04-06

**Authors:** Bo-Ram Jin, Kyung-Sook Chung, Se-Yun Cheon, Minho Lee, Soonjae Hwang, Sam Noh Hwang, Ki-Jong Rhee, Hyo-Jin An

**Affiliations:** 1 Department of Pharmacology, College of Korean Medicine, Sangji University, 83 Sangjidae-gil, Wonju-si, Gangwon-do 220-702, Republic of Korea; 2 Catholic Precision Medicine Research Center, College of Medicine, The Catholic University of Korea, 222, Banpo-daero, Seocho-gu, Seoul, 06591, Republic of Korea; 3 Department of Biomedical Laboratory Science, College of Health Sciences, Yonsei University at Wonju, Wonju-si, Gangwon-do 16493, Republic of Korea

## Abstract

Ulcerative colitis (UC), a type of inflammatory bowel disease (IBD), is a chronic inflammatory disorder of the colon. Although UC is generally treated with anti-inflammatory drugs or immunosuppressants, most of these treatments often prove to be inadequate. Rosmarinic acid (RA) is a phenolic ester included in various medicinal herbs such as *Salvia miltiorrhiz* and *Perilla frutescens*. Although RA has many biological and pharmacological activities, the anti-inflammatory effect of RA in colonic tissue remains unclear. In this study, we investigated the anti-inflammatory effects and underlying molecular mechanism of RA in mice with dextran sulphate sodium (DSS)-induced colitis. In the DSS-induced colitis model, RA significantly reduced the severity of colitis, as assessed by disease activity index (DAI) scores, colonic damage, and colon length. In addition, RA resulted in the reduction of the inflammatory-related cytokines, such as IL-6, IL-1β, and IL-22, and protein levels of COX-2 and iNOS in mice with DSS-induced colitis. Furthermore, RA effectively and pleiotropically inhibited nuclear factor-kappa B and signal transducer and activator of transcription 3 activation, and subsequently reduced the activity of pro-survival genes that depend on these transcription factors. These results demonstrate that RA has an ameliorative effect on colonic inflammation and thus a potential therapeutic role in colitis.

Ulcerative colitis (UC) is a chronic and relapsing inflammatory bowel disease (IBD) involving the large bowel^1^. Maintenance therapy for UC is essential, as untreated UC has 5-times higher relapse rate. In particular, patients with long-standing UC have an increased predisposition to colitis-associated colorectal cancer (CRC)^2^. Furthermore, it has been reported in murine and clinical models that this tumour progression and growth is associated with underlying immune reactions and relative inflammation factors^3^. Although the aetiology of UC has not been clearly demonstrated, there are indications of the involvement of damaged intestinal epithelial tissue and activated immune cells in mucosa and submucosa with excessive production of inflammatory associated cytokines^4^. Additionally, the upregulation of inflammatory-relative proteins, *e.g.* cyclooxygenase (COX)-2 or inducible nitric oxide synthase (iNOS), and commitment of transcription factors, *e.g.* nuclear factor kappa-light-chain-enhancer of activated B cells (NF-κB), signal transducer and activator of transcription (STAT) family, and activator protein 1 (AP-1), have been commonly accepted to be part of the immune deregulation of UC^5^.

Among transcription factors, NF-κB performs a pivotal function in the expression of many genes involved in immune and inflammatory responses, including ones that contribute to IBD. Under normal conditions in intestinal epithelial cells, inactive NF-κB complexes composed of p65 and p50 are present in the cytoplasm by binding to their inhibitory subunit, IκB. In response to stimuli, IκB proteins are phosphorylated, ubiquitinated, and degraded; the ultimate result is the translocation of NF-κB into the nucleus, where it can bind specific DNA binding sites and regulate the transcription of target genes, such as iNOS, COX-2, interleukin (IL)-1β, IL-6, and tumour necrosis factor- α (TNF-α), which are known genes of pro-inflammatory enzymes and cytokines^6^,^7^,^8^. STAT3 is also known to be associated with colonic inflammation, and is activated by a variety of cytokines and growth factors. Activation of STAT3 dimers by progressive phosphorylation ultimately allows for translocates into the nucleus, where it regulates genes involved in apoptosis, cell cycle progression and proliferation, and angiogenesis. Several studies also reported that increased STAT3 phosphorylation at tyrosine residues is found in the murine dextran sulphate sodium (DSS)-induced colitis model, as well as in the epithelial tissue and lamina propria cells of IBD patients^9^,^10^,^11^,^12^.

Rosmarinic acid (RA) is a widespread phenolic ester compound in the plants, particularly those in the Labiatae family of herbs, such as *Rosmarinus officinali, Salvia miltiorrhiza*, and *Prunella vulgaris*. Several studies have reported that RA has various biological and pharmacological activities, including anti-oxidant, anti-mutagenic, and anti-apoptotic activities^13^. Especially, several studies were reported that RA inhibited the induction of allergic-inflammatory reactions and indicated the antisepsis effect mediated by decreasing local and systemic levels of a wide spectrum of inflammatory mediators^14^,^15^. In addition, RA possess anti-inflammatory properties in carrageenan-induced paw oedema rat model and liver ischaemia-reperfusion and thermal injury rat models^16^. Furthermore, it has been reported that RA, as the biologically active element of *Perilla frutescens*, increases the regulatory T cell population in the DSS-induced colitis model^17^. However, there has yet to be a study evaluating the detailed *in vivo* biological efficacies of RA or demonstrating its underlying molecular mechanism in mice with DSS-induced colitis. Therefore, we investigated the anti-inflammatory properties of RA and its associated molecular mechanism involving the regulation of transcription factors in colonic mucosa under acute inflammatory conditions.

## Results

### RA attenuated the progression of DSS-induced colitis in mice

**Figure 1:**
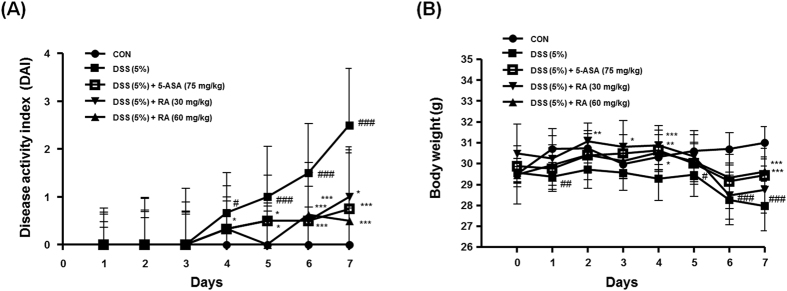
Effect of RA on the development of DSS-induced colitis model. Male ICR (6 weeks of age) mice were administrated with 5% DSS in drinking water (ad libitum) and treated with or without RA (30 or 60 mg/kg/day; p.o), 5-ASA (75 mg/kg/day; p.o.). (**A**) Disease activity index was estimated once per day for 6 days. (**B**) Body weight was measured every day during the experimental period. Values are the mean ± SD (n = 10); ^#^P < 0.05, ^##^P < 0.01, ^###^P < 0.001 vs control group; ^*^P < 0.05, ^**^P < 0.01, ^***^P < 0.001 vs the DSS-induced group; significances between treated groups were determined using ANOVA and Dunnett’s post hoc test.

To estimate the effect of RA on the murine DSS-induced colitis model, mice were administered drinking water with 5% DSS for seven days, and administrated the same with or without RA (30 or 60 mg/kg/day, p.o.) once a day. Symptomatic parameters of colitis, such as body weight loss, rectal bleeding, and severity of stool consistency, were scored from zero to 4 to ultimately obtain the DAI (Table 1). Compared with the control group, the DAI score of the DSS-induced group was considerably increased. However, oral administration of RA (30, 60 mg/kg) significantly ameliorated the severity of rectal bleeding and diarrhoea compared to the DSS-induced group from 4 days (Fig. 1A). As shown in Fig. 1B, the DSS-induced group exhibited significant body weight loss in comparison with the control group, whereas administration of 5-ASA or RA (60 mg/kg) attenuated the progression of DSS-induced body weight loss during experimentation.

### RA prevented the colonic shortening and spleen enlargement in DSS-induced colitis in mice

**Figure 2:**
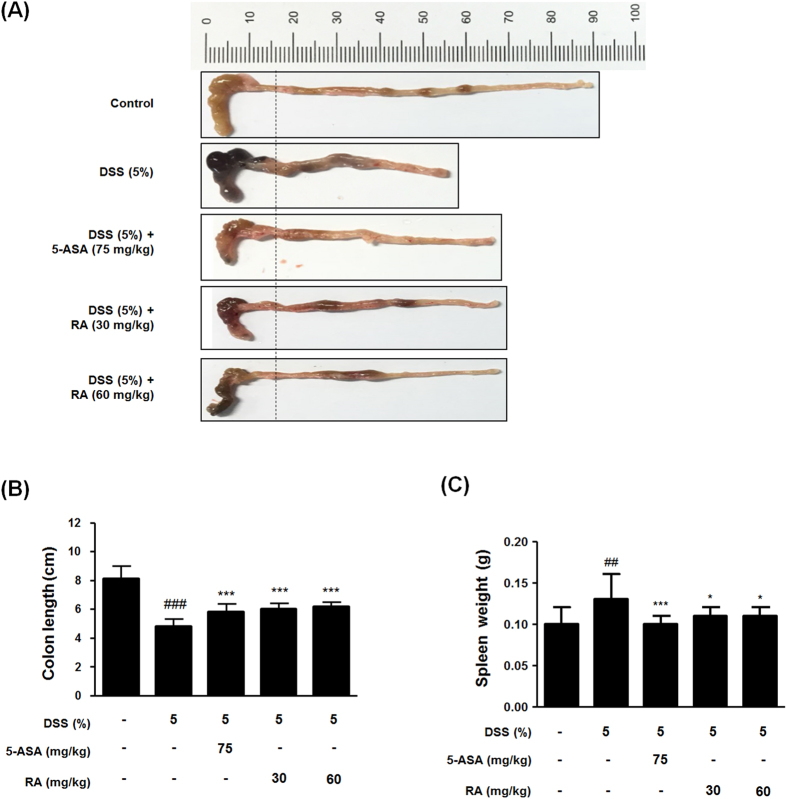
Effect of RA on the symptom of DSS-induced colitis model. (**A**) A representative photograph of colon tissues in each group is provided. (**B**) The colon length was measured when mice were euthanized. (**C**) Spleen weight was measured in each mouse. Values are the mean ± SD (n = 10); ^##^P < 0.01, ^###^P < 0.001 vs control group; ^*^P < 0.05, ^***^P < 0.001 vs the DSS-induced group; significances between treated groups were determined using ANOVA and Dunnett’s post hoc test.

Shortening of colon length in DSS-induced mice is one of the biological markers in the assessment of colonic inflammation^18^. To determine the preventive effect of RA on colitis, we estimated the colon length and spleen weight of the experimental mice. The colon length of the DSS-induced group was shortened compared to that of control group in mice with DSS-induced colitis. In contrast, RA treatment attenuated colonic shortening (Fig. 2A,B). Furthermore, splenic enlargement was observed in mice with DSS-induced colitis, but administration of 5-ASA or RA reduced spleen enlargement (Fig. 2C). These results indicate that the therapeutic effect of RA was similar to that of 5-ASA.

### RA ameliorated inflammation-related symptoms in DSS-induced colitis in mice

**Figure 3:**
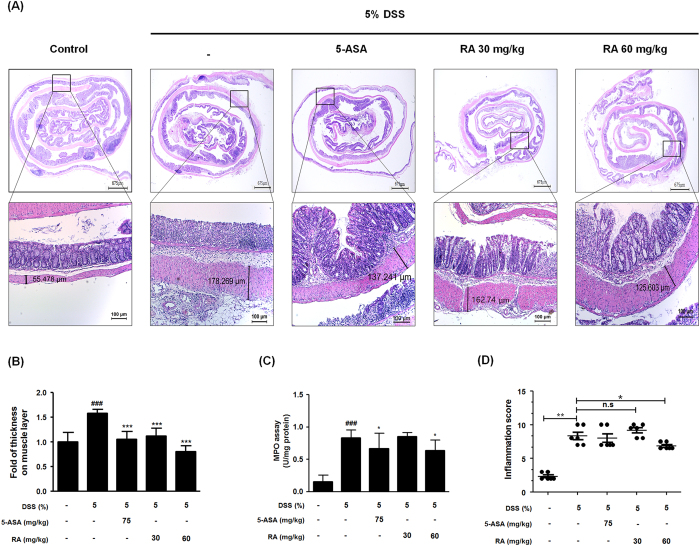
Histological estimation of colorectal tissues and MPO activity. (**A**) Representative portion of colon tissues were stained by H&E. (**B**) Muscle thickness of colon sections were evaluated using LAS software. Stained section was observed by microscope. Magnification x40, x100 inset. (**C**) MPO level in colon tissues was determined. (**D**) Inflammation score in DSS-induced mice was estimated. Values are the mean ± SD (n = 10); ^###^P < 0.001 vs control group; ^*^P < 0.05, ^***^P < 0.001 vs the DSS-induced group; significances between treated groups were determined using ANOVA and Dunnett’s post hoc test.

Colonic inflammation involves the disruption of the architecture of colonic mucosa and ulceration, resulting in the infiltration of inflammatory cells such as inflammatory monocytes and macrophages and thickening of the lamina propria^19^. To investigate mucosal inflammation, we performed H&E staining and demonstrated representative pathological results. As shown in Fig. 3A, histologic alterations in the colon of DSS-induced mice were observed. Treatment with 5-ASA or RA preserved the extension of crypt distortion and ameliorated inflammatory reactions such as mucosal and submucosal infiltrations. Colonic muscle thickness, MPO levels, and inflammation score were measured to evaluate the degree of inflammation in mice with DSS-induced colitis^20^. Compared with the control group, mice in the DSS-induced group had significantly increased colonic muscle thickness. However, administration of RA or 5-ASA suppressed colonic muscle thickening, with inhibitory effect of 60 mg/kg RA greater than that of 5-ASA (Fig. 3B). In accordance with the progression of colonic inflammation, the MPO level in the colonic tissue was elevated. However, administration of 5-ASA or RA (60 mg/kg) significantly decreased MPO levels (Fig. 3C). Additionally, we evaluated the H&E stained images from each group and assigned an inflammatory grade based on Table 2. Overall, the RA (60 mg/kg)-treated group exhibited a greater inhibitory effect of inflammation-related symptom than the 5-ASA-treated group, suggesting that mechanism underlying the protective effects of RA involved a suppression of inflammatory cell infiltration into the colonic mucosa.

### RA attenuated the production of pro-inflammatory cytokines and the expression of COX-2 and iNOS protein in the DSS-induced colitis in mice

**Figure 4:**
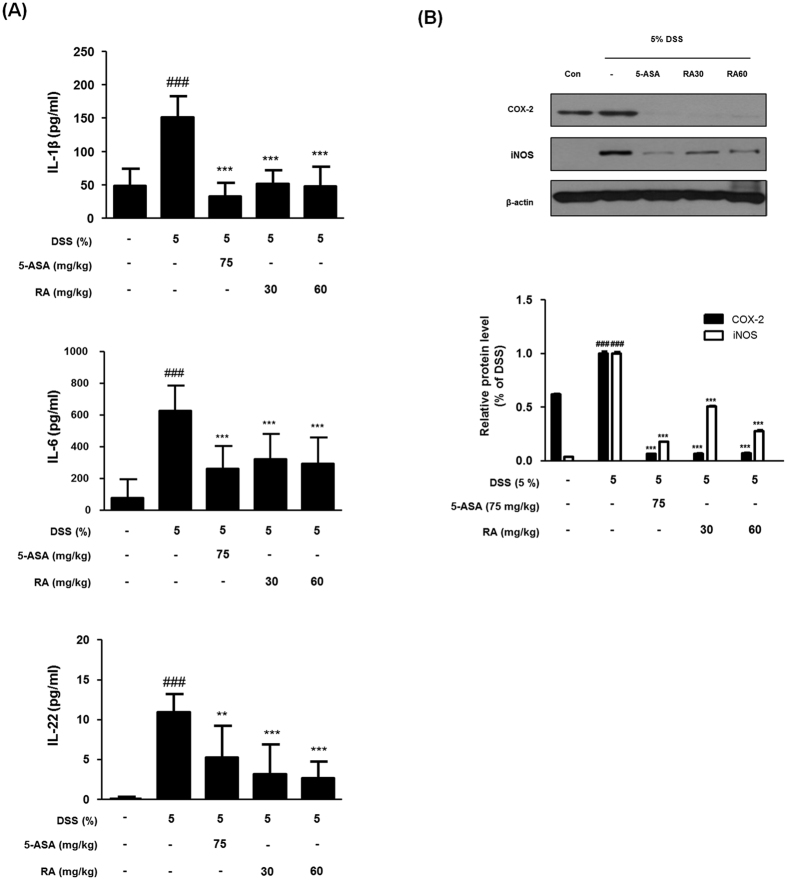
Effect of RA on the pro-inflammatory cytokines and proteins. (**A**) IL-1β, IL-6 and IL-22 were determined by EIA kits. (**B**) The total protein lysates were prepared and estimated for level of COX-2 and iNOS by western blot analysis using specific antibodies. Relative ratio level was determined by densitometric analysis (Bio-rad Quantity One^®^ Software) normalized to β-actin. Values are the mean ± SD (n = 10); ^###^P < 0.001 vs control group; ^**^P < 0.01, ^***^P < 0.001 vs the DSS-induced group; significances between treated groups were determined using ANOVA and Dunnett’s post hoc test.

It was reported that several pro-inflammatory cytokines are implicated in IBD pathogenesis, with anti-inflammatory cytokine therapy are potential targets for remission in patients with IBD^21^. To investigate the effect of RA on the production of pro-inflammatory cytokines such as IL-1β, IL-6, and IL-22 in DSS-induced mice, we analysed cytokine levels in tissues. Compared with the control group, IL-1β, IL-6, and IL-22 production levels in the DSS-induced group significantly increased. However, treatment with RA (30, 60 mg/kg) or 5-ASA markedly attenuated the production of cytokines (Fig. 4A). Next, we evaluated the effect of RA on the expressions of COX-2 and iNOS in colon tissues *via* western blot analysis. DSS markedly induced the expression of COX-2 and iNOS in the colons of mice, while the expression of these proteins was markedly reduced by RA treatment in mice with DSS-induced colitis (Fig. 4B).

### RA suppressed NF-κB activation and expressions of NF-κB–related proteins in DSS-induced colitis mice

**Figure 5:**
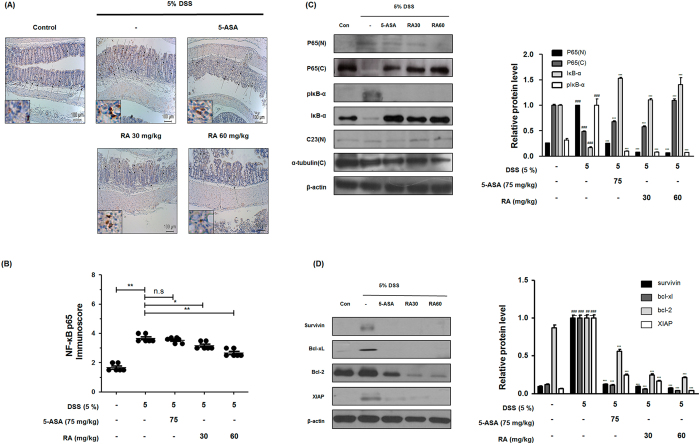
Effect of RA on NF-κB activation and expression of its relative gene products in DSS-induced colitis mice. (**A**) The manifestation and translocation to the nucleus of p65 in colon tissues were observed by immunohistochemistry estimation. (**B**) Immunoscore of p65 in colon of DSS-induced mice was estimated. (**C**) Nuclear (N) and cytosol (C) extracts were prepared from colon tissues on 7 days of DSS administration and translocation of p65 to the nucleus and phosphorylation of IκB were estimated by western blot analysis using specific antibodies. C23 and α-tubulin were used as internal controls. (**D**) NF-κB-related proteins were determined by western blot analysis using specific antibodies. β-actin was used as internal controls. Relative ratio level was determined by densitometric analysis (Bio-rad Quantity One^®^ Software) normalized to internal controls. Values are the mean ± SD (n = 10); ^##^P < 0.01, ^###^P < 0.001 vs control group; ^*^P < 0.05, ^**^P < 0.01, ^***^P < 0.001 vs the DSS-induced group; significances between treated groups were determined using ANOVA and Dunnett’s post hoc test.

NF-κB signalling is a key process during inflammation and thus constitutes an attractive target for anti-inflammatory interventions^8^. To clarify the molecular mechanisms of RA, we evaluated its effect on NF-κB activation in DSS-induced colitis mice. As shown in Fig. 5A, IHC analysis demonstrated that RA markedly suppressed the protein expression of NF-κB p65 in the inflamed mucosa and inhibited translocation of NF-κB p65 to the nucleus. In addition, immunoreactivity score of p65 was assessed in the representative pictures of each group according to Table 3. The mice in the DSS-induced group had a markedly increased score compared to that of the control group; the score of the RA-treated mice was significantly decreased in comparison (Fig. 5B). In western blot analysis, the colons of DSS-administered mice had a sharp increase in the translocation of p65 from cytosol to the nucleus. In contrast, RA treatment markedly inhibited the DSS-induced nuclear translocation of p65 in colonic tissues (Fig. 5C). Moreover, we determined that RA inhibits the phosphorylation and degradation of IκB in the colonic tissue of DSS-induced mice. We also found that DSS increased the expression levels of NF-κB-related proteins, such as survivin, Bcl-2 family proteins, and XIAP, whereas RA treatment reduced these increases in the colonic tissue of DSS-induced mice (Fig. 5D).

### RA inhibited STAT3 activation and expressions of STAT3-related proteins in DSS-induced colitis in mice

**Figure 6:**
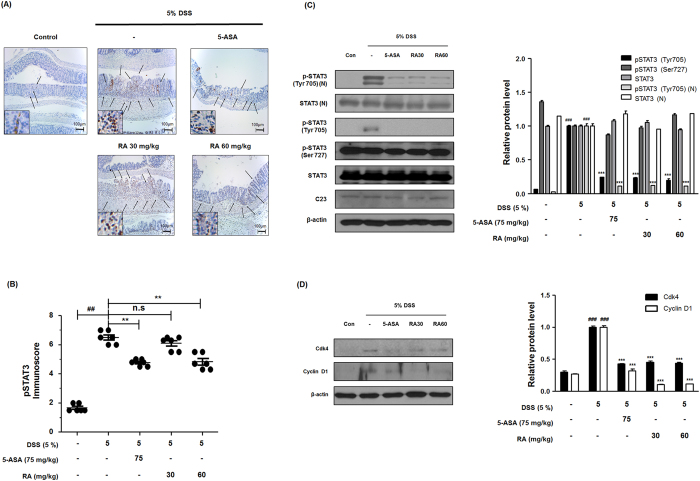
Effect of RA on constitutive activation of STAT3 and its relative gene products in DSS-induced colitis mice. (**A**) The manifestation and translocation to the nucleus of pSTAT3 (Tyr705) in colon tissues were performed by immunohistochemistry. (**B**) pSTAT3 (Tyr705) immunoscore in DSS-induced mice was estimated. (**C**) Nuclear (N) and cytosol (**C**) extracts were prepared from colon tissues on 7 days of DSS administration and the phosphorylation and nuclear translocation of pSTAT3 were estimated by western blot analysis using specific antibodies. C23 and β-actin were used as internal controls. (**D**) The expression of STAT3 target protein was determined by western blot analysis using specific antibodies. Relative ratio level was determined by densitometric analysis (Bio-rad Quantity One^®^ Software) normalized to internal controls. Values are the mean ± SD (n = 10); ^##^P < 0.01, ^###^P < 0.001 vs control group; ^***^P < 0.001 vs the DSS-induced group; significances between treated groups were determined using ANOVA and Dunnett’s post hoc test.

STAT3 has now been found to be associated with inflammation, cellular transformation, survival, proliferation, and invasion^9^. To investigate the cellular mechanisms of RA that are responsible for DSS-induced colitis, we examined the effect of RA on STAT3 activation in DSS-induced colon tissues. As shown in Fig. 6A,B, immunohistochemistry (IHC) results reveal that treatment with RA significantly suppresses the expression of pSTAT3 and its translocation to the nucleus in the colonic tissue of mice with DSS-induced colitis. Furthermore, western blot analysis indicates that RA inhibits only STAT3 phosphorylation at the tyrosine-705 residue in the nuclear fraction and whole protein, but does not affect the phosphorylation of STAT3 at the serine-727 residue (Fig. 6C). We also investigated whether RA affects the expression of STAT3-related proteins in colonic tissue. As shown in Fig. 6D, we observed that DSS increases the expression of STAT3-related proteins, *i.e.* Cdk4 and cyclin D1, whereas RA inhibits these increases in the colons of mice with DSS-induced colitis.

## Discussion

IBD is a class of multifactorial, chronic disorders of the gastrointestinal tract; it is composed of two chief types, UC and Crohn’s disease (CD). IBD affects millions of people worldwide, and is significantly associated with colorectal cancer (CRC) risk, whose incidence is now rapidly increasing in previously risk-free continents, such as Asia and South America, due to westernized diets^22^. Although UC and CD have similar clinical pathologic characteristics, they also have marked differences, such as disease course and immunological genotype^23^,^24^.

Regarding the chemotherapeutic regimes used to treat IBD, 5-ASA is considered one of the cornerstone anti-inflammatory drugs, and is mainly used in the treatment of UC. 5-ASA, also known mesalazine, yields immunosuppressive effects such as prostaglandin restriction and pro-inflammatory cytokine inhibition. As the active moiety of sulfasalazine, this medication has been commonly used to treat mild to moderate ulcerative colitis for over 60 years. However, 5-ASA can have adverse effects, including headache, abdominal pain, hair loss, and allergic reactions^25^,^26^. Therefore, the development of alternative anti-inflammatory regimens with decreased side effects is necessary to improve patient quality of life. Recently, the immunomodulatory agents originating from herbal medicine represent a promising approach for UC therapy, as shown by the variety of clinical trials and experimental studies currently underway^27^. RA, an active ingredient from various herbs, has been reported to possess anti-oxidant, anti-inflammatory, and anti-tumour functionality, as well as attenuate allergic diseases and the development of Alzheimer’s disease^28^. Thus, we can hypothesize that RA could also have a beneficial effect on UC.

The present study was designed to evaluate the effect of RA on a murine DSS-induced colitis model. This model is often used to interpret the complex and varied causes of colitis, and mimics many manifestations of human UC, including disruption of the epithelium, mucosal ulceration, as well as systemic symptoms such as shortening of the colon, diarrhoea, hemafecia, anaemia, and eventually death^29^. Acute colitis is commonly induced by administration of 2–5% DSS for 4–9 days^30^. In this study, mice were administered 5% DSS in drinking water *ad libitum* for 7 days with or without RA (30, 60 mg/kg/day p.o. up to 7 days). 5-ASA (75 mg/kg/day p.o.) was used as a positive control.

The measurement of body weight loss is a standard way to evaluate disease progression in the DSS-induced colitis model. Loss in weight and archorrhagia are connected with colon shortening. Spleen enlargement is associated with splenic macrophage infiltration after beginning DSS administration^18^. In this study, we observed a DSS-induced DAI increase after evaluation of parameters such as extent of weight loss, stool consistency, and hematochezia. In contrast, RA treatment was found to significantly decrease DAI and suppress weight loss (Fig. 1A,B). In addition, RA suppressed colon length shortening and spleen enlargement in DSS-induced mice (Fig. 2A,B). It has been reported that the DSS-induced colitis model can represent several histopathological features of UC, such as mucosal erosion, loss of intestinal crypts, and ulceration. In the present study, RA significantly ameliorated inflammatory cell infiltration compared with the DSS-induced colitis model (Fig. 3A). Furthermore, bowel wall thickness associated with inflammation was suppressed with RA treatment (Fig. 3A,B). MPO is an enzyme that is detected mainly in neutrophils, but also to a small degree in monocytes and macrophages. Thus, MPO activity reflects the degree of neutrophil infiltration, so it can serve as a marker of acute inflammation^31^. Infiltration of macrophages into inflamed mucosa has been observed in IBD patients^32^. Our study demonstrated that RA treatment (60 mg/kg) significantly decreases inflammation score. Based on these data, we conclude that RA has therapeutic effects in mice with DSS-induced colitis.

Our results also indicate that RA inhibits the induction of COX-2 and iNOS expressions and the production of pro-inflammatory cytokines such as IL-1β, IL-6, and IL-22. In fact, IBD patient specimens have been associated with excessive concentrations of pro-inflammatory cytokines and enzymes regulated by the activation of transcriptional factors such as NF-κB and STAT3^8^,^33^. In various studies, it has been reported that NF-κB and STAT3 activation promotes cell proliferation, survival, and angiogenesis, and up-regulates the expression of target genes including cyclin D1 and Bcl-xL family^34^. The NF-κB cascade is linked closely with the expression of Bcl-xL, Bcl-2, XIAP, and regulatory cell cycle genes, such as IL-6 and cyclin D1^35^. Moreover, the stimulation of IL-6 also leads to constitutive activation of STAT3, which result in high expression of Bcl-xL^36^. STAT3 and NF-κB cooperatively regulate a number of target genes and also synergistically control relative cytokines and chemokines. In addition, it has been reported that STAT3 directly interacts with p65 and p50, facilitating NF-κB recruitment to STAT3 promoters and vice versa. STAT3 can adjust RelA post-translationally by recruitment of the acetyltransferase p300, mediating NF-κB acetylation and prolongation of its nuclear retention^37^. Moreover, overexpression of the serum amyloid A, one of the sensitive marker of acute-phase responses, is closely related to the formation of a complex with STAT3, NF-κB p65 during inflammation^38^. The activation and crosstalk between STAT3 and NF-κB is commonly outcome of chronic inflammation and tumour microenvironments, especially with inflammatory cells that infiltrate tumours. In these regards, the NF-κB and STAT3 pathway has emerged as a potential therapeutic target in colonic disease^39^. In our study, phosphorylation of the STAT3 Tyr705 in particular was associated with colitis of DSS-induced mice, but not STAT3 Ser727. Phosphorylation of STAT3 at Tyr705, not Ser727 in the DSS-colitis model correlates with previous studies^40^. STAT3 activity is absolutely dependent on tyrosine phosphorylation, while serine phosphorylation appears to be associated with maximal activation^41^. The serine residue of STAT3 can be phosphorylated independent of JAK activity and STAT3 tyrosine phosphorylation^42^. In this study, we demonstrated the inhibitory effect of RA on the constitutive activation and translocation to nucleus of NF-κB and STAT3 Tyr705 in mice with DSS-induced colitis. These results suggest that RA could ameliorate DSS-induced acute colitis by inhibiting the NF-κB and STAT3 Tyr705 signalling pathways.

In summary, our study shows that RA significantly ameliorates systemic symptoms in a murine DSS-induced colitis model and suppresses expression of pro-inflammatory cytokines and inflammatory mediators through regulation of NF-κB and STAT3 activation. We therefore suggest that RA deserves further consideration as a potential therapeutic for the treatment of inflammatory diseases such as colitis.

## Materials and Methods

### Chemicals and reagents

DSS was purchased from MP Biomedicals (Santa Ana, California, USA). IL-1β and IL-6 enzyme immunoassay (EIA) kits were purchased from BD Biosciences (San Diego, California, USA), and the EIA kit for IL-22 was purchased from R&D systems (Minneapolis, Minnesota, USA). Primary antibodies for p65, iNOS, COX-2, STAT3, IκB-α, cyclin D1, β-actin, pIκB-α, XIAP, survivin, XIAP, Bcl-2, Bcl-xL, and Cdk-4 are purchased from Santa Cruz Biotechnology, Inc. (Dallas, Texas, USA), and the primary antibodies for p-STAT3 (Ser727) and p-STAT3 (Tyr705) were purchased from Cell Signalling Technology (Danvers, Massachusetts, USA). Peroxidase-conjugated secondary antibodies were purchased from Jackson ImmunoResearch, Inc. (West Grove, Pennsylvania, USA). Dulbecco’s modified eagle’s medium (DMEM), foetal bovine serum (FBS), penicillin, and streptomycin were purchased from Gibco (Waltham, Massachusetts, USA). 5-Aminosalicylic acid (5-ASA), rosmarinic acid, and all other chemicals were purchased from Sigma-Aldrich Co. (St. Louis, Missouri, USA).

### Experimental animals

Male ICR mice (n = 50; 6-week-old) were purchased from Raon Biolink (Yonginsi, Republic of Korea). All mice were randomly allocated to five per cage and housed under standard conditions (constant temperature of 20 ± 5 °C, indoor relative humidity of 55 ± 10%, 12 h dark/light cycles). All mice were fed standard laboratory chows (Research Diets, New Brunswick, NJ, USA). All animal experiments were approved by the Committee for Animal Care and Use of Sang ji University (No. 2016-12, Wonju-si, Republic of Korea) and conducted according to an animal protocol.

### Induction of colitis

Experimental colitis was induced by giving mice drinking water *ad libitum* containing 5% (w/v) DSS for 7 days. Mice of each of the groups were monitored carefully every day to confirm that they had consumed an approximately equal volume of water containing DSS. For each experiment, the mice were divided into five experimental groups (n = 10/group). The first group was kept as the vehicle-treated control, and the second group was given drinking water with DSS only during the experimental period. The other three groups consisted of mice receiving 5% DSS who were administrated 5-ASA (75 mg/kg/day p.o.) or rosmarinic acid (30 or 60 mg/kg/day p.o.) daily for 7 days, according to the experimental design. All materials were dissolved in a vehicle of 0.9% saline. Control groups were given the vehicle daily for 7 days as appropriate. Administration of each drug was initiated simultaneously with the DSS treatment.

### Evaluation of disease activity index (DAI)

Body weight, stool consistency, and gross bleeding were recorded daily. Disease activity index (DAI) was determined by combining the scores for (i) body weight loss, (ii) stool consistency, and (iii) gross bleeding, divided by 3. Each score was determined as follows: change in body weight loss (0: none, 1: 1–5%, 2: 5–10%, 3: 10–20%, 4: > 20%), stool blood (0: negative, 1: +, 2: ++, 3: +++, 4: ++++), and stool consistency (0: normal, 1 and 2: loose stool, 3 and 4: diarrhoea). Body weight loss was calculated as the percentage of the difference between the original body weight (day 0) and the body weight on any particular day (Table 1). At the end of experiment, all mice were sacrificed and the large intestines were separated from the vermiform appendix to the anus. The colon length was measured between the caecum and proximal rectum. The spleens were dissected and their weights measured immediately.

### Histopathology

The resected mice colon tissues were fixed immediately in 10% formalin and embedded. For histopathological analysis, tissue samples were sectioned (5 μm) and stained with haematoxylin and eosin (H&E) and periodic acid-Schiff (PAS). Both of the histologic processes were described previously^43^.

### Measurement of myeloperoxidase activity (MPO) and cytokine production

Colon tissues were washed with DMEM medium containing 0.2% FBS, streptomycin, and penicillin, and cut into smaller pieces. Afterwards, 0.5 cm of the tissue was placed in a 24-well plate filled with 1 ml DMEM medium containing 0.2% FBS, streptomycin, and penicillin, and incubated for 24 h at 37 °C in 5% CO_2_. The cell-free culture supernatants of the colon tissue were used to measure MPO activity and production of cytokines. Neutrophil sequestration in the colon was quantified by measuring tissue MPO activity. To estimate MPO activity, tissue samples were thawed and homogenized in 0.05 M phosphate buffer (pH 6) containing 0.5% (w/v) hexadecyltrimethylammonium bromide. The suspension was centrifuged (3,000 rpm, 20 min, 4 °C), and the supernatant was used for MPO assay. The reaction mixture consisted of the supernatant, 0.003% H_2_O_2_ (Sigma-Aldrich), O-dianiside in 0.05 M phosphate buffer (pH 6), and 0.5% HTAB. This mixture was incubated at 37 °C and terminated at 10 min. The absorbance was measured at 450 nm. The results were quantified as the amount from 10 min absorbance minus the amount from zero min absorbance, and expressed as unit per milligram of protein. In addition, the levels of IL-1β, IL-6, and IL-22 produced in the culture media were quantified using EIA kits, according to the manufacturer’s instructions.

### Inflammation score

The inflammation score was evaluated based on Table 2. Inflammation was graded as follows: mucosal epithelial cell 1, prolonged epithelial cell or crypt; 2, destruction of barrier; 3, ulcer (30% < loss < 60%); 4, ulcer (loss > 60%), mucosal immune cell 1, infiltration (mild); 2, infiltration (moderate); 3, infiltration (severe) and submucosa’s immune cell 1, infiltration (mild); 2, infiltration (moderate); 3, infiltration (severe).

### Immunohistochemistry

All IHC was performed on formalin-fixed, paraffin-embedded samples. Paraffin blocks were sectioned to 5-μm thickness. Afterwards, poly-L-lysine-coated slides were used to promote adhesion of the paraffin-section to the slides, which were then dried. The dried slides were de-paraffinized, and antigen retrieval was performed by automated antigen retrieval machine for 20 minutes in cell condition solution (Ethylenediaminetetraacetic acid pH 9.0). Sections were blocked for 1 h with 15–20% normal goat serum (Gibco Life Technologies, Grand Island, NY, USA), prior to incubation with primary antibody for 2 h at room temperature or overnight at 4 °C. Secondary rabbit antibodies were used to detect primary antibodies, followed by streptavidin-tagged horseradish peroxidase (Ventana Medical Systems, Tucson, USA). Diaminobenzidine (DAB, Sigma-Aldrich, St. Louis, Missouri, USA) was used to induce signalling, and bluing reagent (Ventana Medical Systems, Tucson, USA) was used as a counterstain. Images of IHC slides were visualized by optical microscopy (Leica, Wetzlar, Germany) and rendered using Leica software. For IHC, p-STAT3 (Tyr705) and NF-κB p65 antibodies were used.

### Western blot analysis

Colon tissues from each group were thawed and homogenized using the protein lysis buffer Pro-prep^TM^ (Intron biotechnology Inc, Kyungki-Do, Republic of Korea). Protein extracts were isolated from colon tissue. Protein samples (30 μg each) were separated on an 8–12% sodium dodecyl sulphate-polyacrylamide gel. After electrophoresis, proteins were transferred to polyvinylidenedifluoride membranes. The membranes were blocked in 2.5–5% skim milk for 30 min and incubated overnight with specific primary antibodies in Tris-buffered saline (TBS) containing 0.1% Tween 20 at 4 °C. Primary antibody was removed by washing the membranes 3 times in TBS-T buffer, and incubated for 2 h with horseradish peroxidase-conjugated secondary antibody (1: 2500) at 25 °C. After washing three times in TBS-T, immuno-detection bands were reacted with ECL solution (Ab signal, Seoul, Republic of Korea) and manifested on X-ray film (Agfa, Belgium).

### Statistical analyses

Results are expressed as the mean ± S.D. of triplicate experiments. Statistically significant values were determined using ANOVA and Dunnett’s post hoc test, and *p*-values of less than 0.05 were considered statistically significant.

## Additional Information

**How to cite this article:** Jin, B.-R. *et al*. Rosmarinic acid suppresses colonic inflammation in dextran sulphate sodium (DSS)-induced mice via dual inhibition of NF-κB and STAT3 activation. *Sci. Rep.* **7**, 46252; doi: 10.1038/srep46252 (2017).

**Publisher's note:** Springer Nature remains neutral with regard to jurisdictional claims in published maps and institutional affiliations.

## Figures and Tables

**Table 1:** Evaluation of disease activity index (DAI).

DAI score	Wt loss (%)	Stool consistncy	Occult/Gross bleeding
0	None	Normal	Normal
1	1–5		
2	5–10	Loose stools	Hemoccult positive
3	10–20		
4	> 20	Diarrhea	Gross bleeding

**Table 2:** Score of histopathological colitis.

Histological parameters		Description	Score
Mucosa	Epithelial cell	Prolonged epithelial cell or crypt	1
		Destruction of barrier	2
		Ulcer (30% < loss < 60%)	3
		Ulcer (loss > 60%)	4
	Immune cell	mild Infiltration	1
		moderate Infiltration	2
		severe Infiltration	3
Sub- mucosa	Immune cell	mild Infiltration	1
		moderate Infiltration	2
		severe Infiltration	3

**Table 3:** Score of immune-reactivity.

Reigion	Description	Score
Non-inflammatory region	Mild	1
	Moderate	2
	Intense	3
	Severe	4
Inflammatory region	Mild	1
	Moderate	2
	Intense	3
	Severe	

Severe Score of Inflammatory region is 4.
